# Barriers and facilitators to implementation of direct fruit and vegetables provision interventions in kindergartens and schools: a qualitative systematic review applying the consolidated framework for implementation research (CFIR)

**DOI:** 10.1186/s12966-022-01246-8

**Published:** 2022-01-31

**Authors:** Biljana Meshkovska, Daniel A. Scheller, Janine Wendt, Hannah Jilani, Marie Scheidmeir, Jan M. Stratil, Nanna Lien, on behalf of the PEN Consortium

**Affiliations:** 1 Department of Nutrition, University of Oslo, Oslo, Norway; 2 Division of Sports and Rehabilitation, Department of Internal Medicine II, Ulm University Medical Centre, Ulm, Germany; 3 Institute of Public Health and Nursing Research - IPP, University of Bremen and Epidemiological Methods and Etiological Research, Leibniz Institute for Prevention Research and Epidemiology - BIPS, Bremen, Germany; 4 Department of Psychology, Johannes Gutenberg-University Mainz, Mainz, Germany; 5 Institute for Medical Information Processing, Biometry, and Epidemiology – IBE, Chair of Public Health and Health Services Research, LMU Munich, Munich, Germany

**Keywords:** Fruit, Vegetables, Intervention, Implementation, Barrier, Facilitator, Determinant, Consolidated framework for implementation research

## Abstract

**Background:**

Although children’s intake of fruit and vegetables has seen a recent rise, almost half of adolescents do not eat even one piece of fruit or vegetables per day. One way to address this problem is through interventions that provide fruit and vegetables directly to children in kindergartens and schools. For such interventions to meet their intended goals, what is important to consider in addition to impact is implementation. Our objective is to systematically review qualitative results reporting on the determinants (barriers and facilitators) to implementation of interventions that entail direct provision of fruit and vegetables in kindergarten and school settings and conduct a framework analysis of those results using the Consolidated Framework for Implementation Research (CFIR).

**Methods:**

A systematic search was designed and run in November 2019 for: MEDLINE (Ovid), Embase (Ovid), PsychINFO (Ovid), ERIC (Ovid), Cochrane Library Reviews and Cochrane Library Trials. A keyword search of the journal *Implementation Science* was completed. Screening of titles and abstracts (*n* = 5427) and full texts (*n* = 227), led to 14 included articles. Coding and analysis were done using the framework method and CFIR.

**Results:**

The following CFIR constructs were found relevant: 1) *intervention characteristics* domain: ‘design quality and packaging’, ‘adaptability’ ‘cost’; 2) *outer setting:* ‘cosmopolitanism’, ‘external policy and incentives’ ‘patients’ needs and resources’; 3) *inner setting:* ‘implementation climate’, ‘readiness for implementation’ and ‘structural characteristics’; 4) *characteristics of individuals*: ‘individual stage of change’, ‘knowledge and beliefs about the intervention’ 5) *process:* ‘engaging’, ‘executing’ and ‘reflecting and evaluating’. The review stresses the dual role of parents as both supporting the implementation and targets of the intervention, which could have implications for the design and implementation of future fruit and vegetables interventions. Positive child perceptions of the value of the intervention and perceived behavior change due to the intervention were reported as relevant facilitators to implementation across several studies, and should be taken into consideration in future design efforts.

**Conclusions:**

CFIR offers a systematic way to identify and organize barriers and facilitators to implementation of interventions in the kindergarten and school setting. Revisions are encouraged to allow adequate space for perceptions of various implementation actors and the target group.

**PROSPERO registration:**

CRD42020167697.

**Supplementary Information:**

The online version contains supplementary material available at 10.1186/s12966-022-01246-8.

## Introduction

A higher fruit and vegetables consumption is significantly associated with lower risk of all-cause mortality [1]. Nonetheless, current global consumption levels of fruit and vegetables fall far short of the five a day mark and World Health Organization (WHO) recommendation to eat 400 gr of fruit and vegetables per day [2]. Intake in children has seen a recent rise, nonetheless, 48% of adolescents do not have even one piece of fruit or vegetables daily [3].

One way to address this issue is through interventions that provide fruit and vegetables directly to children in kindergartens and schools, as the settings where many children may be reached. In the context of this article, interventions are understood as any policy, programme or environmental change that aims to promote certain health behaviors [4]. A systematic review and meta-analysis found that interventions in the school setting which directly provide fruit and vegetables to children, increase fruit intake by 0.27 servings and vegetables intake by 0.04 servings per day [5]. These findings were included in a recent umbrella review that concluded there is evidence showing effectiveness of interventions in the school settings on fruit and vegetables consumption [6]. However, for such school based interventions to meet their intended goals, what is important to consider is not merely their content, but also their implementation. Research to date has clearly shown that the level of implementation of any intervention has a direct impact on intended intervention outcomes [7].

Research in the field of implementation science has made significant progress, laying the groundwork in regard to theory [8]. In particular, when studying barriers and facilitators to implementation, various determinant frameworks have been developed, and guidance on which to select and how to use them has also been offered [9, 10]. One of the most comprehensive and widely used determinant frameworks is the Consolidated Framework for Implementation Research (CFIR) [9, 11]. As a determinant framework, CFIR specifies constructs (independent variables) which may influence processes and/or implementation outcomes (dependent variables) [12]. CFIR consists of five domains (intervention characteristics, outer setting, inner setting, characteristics of individuals and process), 26 constructs and 13 sub-constructs [11]. The application of CFIR, when investigating determinants of implementation would not only ensure that no barriers and facilitators are missed, but offer the possibility to compare findings across different studies [13]. A recently published systematic review summarized process evaluations of fruit and vegetables provision interventions in school settings, but limited its scope to interventions where fruit and vegetables were only offered as snacks, and did not use an implementation science based framework for synthesis of results [14].

Our objective is to systematically review qualitative results reporting on the determinants (barriers and facilitators) to implementation of interventions that entail the action of direct provision of fruit and vegetables in kindergarten and school settings and conduct a framework analysis of those results using the CFIR.

## Methods

A protocol for this systematic review has been published in PROSPERO (registration number: CRD42020167697).

### Search strategy

The search strategy was developed by an advisor at the Medical Library, University of Oslo for the following databases: MEDLINE (Ovid), Embase (Ovid), PsychINFO (Ovid), ERIC (Ovid), Cochrane Library Reviews and Cochrane Library Trials (for documentation of full literature search see Additional file 1). Various combinations of the following keywords were used: 1) fruit, vegetables, 2) school (nursery, kindergarten, high, middle, primary), 3) policy, health promotion, intervention, scheme 4) program evaluation, implementation science, process evaluation. The search of the databases was run November 2019, and produced 5240 hits (after deduplication). A keyword search of the journal *Implementation Science* was completed July 2020, producing 156 records. In addition, a manual search was completed of reference lists from 30 studies included in the Micha et al., 2018 systematic review, that were identified as reporting on the impact of direct fruit and vegetables provision interventions [5]. A final manual search was conducted for peer reviewed articles reporting on implementation of the direct fruit and vegetables provision interventions, reported in the noted 30 records by Micha et al. [5].

### Inclusion criteria

We define direct provision fruit and vegetables intervention, as an intervention that would promote the intake of fresh and unprocessed fruit and vegetables by children, free of charge or subsidized, in kindergartens, primary and secondary school environments. Interventions which provide fresh fruit and vegetables on school property, at any time during the school day–outside of usual school meals and/or during usual school meals – are included (for full definition of direct provision intervention please refer to Additional file 2).

**Fig. 1:**
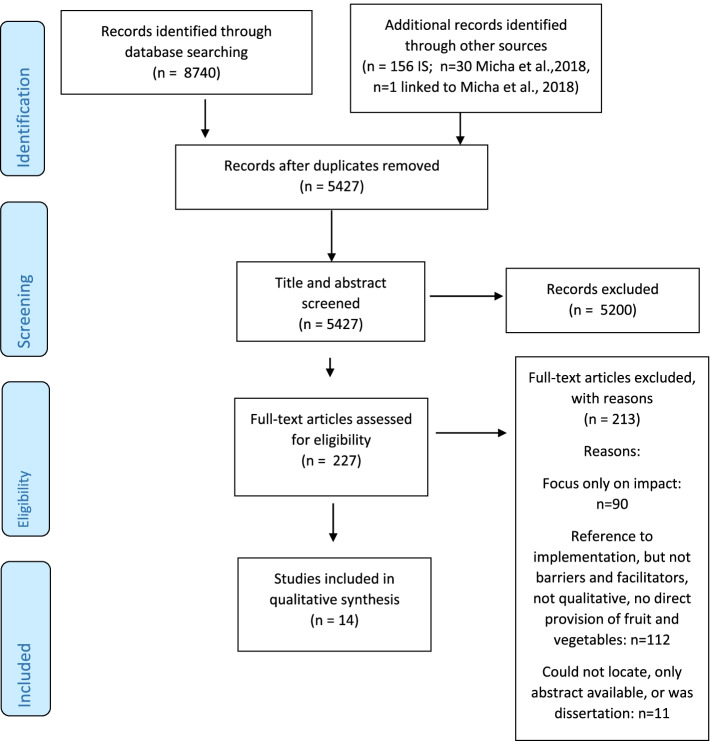
PRISMA flow diagram [64]

Title and abstract screen of the total 5427 records was done independently by two reviewers (B.M. and H.J.) and conflicts were resolved through discussion and consensus. Records that evaluated the impact and/or implementation of interventions providing fresh fruit and vegetables to children on school property, at any time during the school day were included for full text screening. Reviews, study protocols, comments, editorials and conference abstracts were excluded. A total of 5200 records were excluded, leaving 227 records for full text assessment. Quantitative studies reporting only on impact and/or implementation outcomes were excluded, as such results were not sufficient to identify barriers and facilitators to implementation. Full text eligibility evaluation was conducted independently by two teams of reviewers (B.M. and as a team—D.A.S. and J.W.) and conflicts were resolved through discussion and consensus. A total of 213 records were excluded with reason, leaving a final number of 14 articles to be included as part of this review. As 14 peer reviewed articles were identified for inclusion, dissertations and records which were not peer-reviewed were excluded. Grey literature was not searched and included (Fig. 1).

### Quality assessment

For the purpose of quality assessment, two checklists designed specifically to evaluate qualitative research were combined. We took as the basis of our assessment the ten questions of the Critical Appraisal Skills Programme (CASP) [15]. However, we found that the list was lacking in that it did not ask for an assessment of whether articles were based on a ‘theoretical framework’, nor whether articles sufficiently covered the necessary ‘references’ from the field of study – both topics included in the ‘Guidelines for authors and reviewers of qualitative studies’ by Malterud [16]. Thus, these two questions were added to the core ten questions of CASP to compose a combined list of 12 points on which the 14 articles of this review were assessed.

### Characteristics of primary studies

The 14 articles included in this review are based on research conducted in the United States (9), Canada (2), Denmark (2) and Australia, published in the period 2011–2019. For all studies, the school (with one focusing on preschool) was the main setting where implementation of the intervention/program took place. However, three articles also looked at the wider community context.

The main methods for data collection were 1) individual face to face or telephone interviews, 2) focus groups and 3) observation. In two studies, questionnaires were used to collect qualitative data: Lin et al. [17] used qualitative data from one open ended question collected from 3811 children, and Hector et al. [18] used questionnaires to collect qualitative data from 55 teachers, and 4 key contacts in participating schools. Overall, individual interviews and focus groups were conducted with the following target groups: 473 children, 165 school based implementing actors (predominantly teachers-82 and principals-34), 64 parents, 34 suppliers/farmers and 212 other (nutrition practitioners, community residents and experts). The primary method used with children was focus groups, whereas individual interviews were dominant with the remaining target groups of the studies (for full description of interventions, methods and sample size see Tables 1 and 2).

### Data extraction and analysis

For the purpose of data extraction, we have followed the method of Malterud [19–22], that considers data extraction as the ‘process by which we single out and collect relevant information from the included studies’ [19]. Once data extraction was completed and verified, coding and analysis was conducted using the framework method and CFIR [23]. For the purpose of this review, in categorizing the extracted text segments, we have used the definitions for CFIR terms provided on the CFIR website [24]. For full information on the step-by-step process, please refer to Additional File 1.

## Results

### Description of included interventions

The 14 papers included in this review [17, 18, 25–36] are based on 12 interventions—two papers are based on the Boost intervention [26, 36] while one paper refers to the pilot, and one to the full roll out of the Northern fruit and vegetable program in Ontario, Canada [25, 30]. The 12 interventions can be broadly categorized into three types. The **first** are interventions with a limited time frame, implemented once and discontinued upon completion. In addition to providing fruit and vegetables, these interventions have an educational component, and may entail actions that aim to involve groups other than children, such as parents. These are the Boost intervention, Denmark [26, 36]; SnaX intervention, USA [32]; Cooking up Diversity intervention, USA [29] and the supplementary pilot intervention as part of Crunch and Sip, Australia [18]. The **second** type of interventions are those based on national level government policy, that once introduced are then continued on a yearly basis. The main purpose of these interventions is the provision of fresh fruit and vegetables, and they may or may not have additional components. These are the Northern fruit and vegetable program, Canada [25, 30] and the United States Department of Agriculture Fresh Fruit and Vegetable Program, USA [17, 31]. Finally, the **third** type of interventions are farm to school, USA [27, 33–35] and garden programs, USA [28]. They are primarily characterized by their flexibility in the design of intervention components and implementation but are particular in their involvement of the wider community where they are put into practice. For example, one of the core components of farm to school programs is the use of locally grown produce [27].

**Table 1:** Characteristics of primary studies

**Author, year**	**Intervention description (as described in article, with small edits and summaries for flow of read)**	**Land**	**Data collection method**	**Sample size and target group**	**Context**
**Aarestrup et al. (2014)** [26, 36]	The Boost intervention consists of five parts: 1) provision of free fruits and vegetables 2) creating a pleasant eating environment 3) class—based curriculum activities 4) inclusion of parents through meetings at school and newsletters 5) information sheets distributed to sports and youth clubs. The intervention lasted for 9 months and was implemented in 2010/2011. (p.2)	Denmark	focus group interviews class observations telephone interviews	6 schools class observations—no estimate of number of persons observed 111 students (13 years old) 13 teachers 18 suppliers	Community
**Bateman et al. (2014)** [33]	Farm to school programs consist of three parts: 1) local or regional food procurement by schools and preschools 2) nutrition and agricultural education 3) student engagement activity. Fruits and vegetables can be served as snacks, but also as part of a salad bar, or a hot meal at the school. There is variation in how the program is designed and implemented across the United States. (p.49)	USA (Wisconsin)	phone interviews	10 producers (farmers) 5 distributors	Community
**Bogart et al. (2018)** [32]	The SNaX intervention consists of two parts: 1) school wide-food environment changes 2) school wide social marketing. In the context of the first part (environmental changes) sliced and bite sized fruits and vegetables were freely distributed. Students (in the role of 'Student Advocates') and teachers were implementing actors in the school setting. Teachers distributed lists of proposed activities to students, which they could complete at home with their parents. Intervention was implemented in 2014/2015 school year. (p. 725)	USA	interviews focus groups	16 teachers 16 principals 14 cafeteria managers 154 students	Schools
**Bouck et al. (2011) **[30]	The Norther fruit and vegetable pilot program consists of two parts: 1) free distribution of fruits and vegetables 2) more intensive nutrition education. Fruits and vegetables were distributed three times per week, in class. The program was implemented December 2006 to May 2007. (p.15)	Canada (Ontario)	qualitative interviews	28 stakeholders: -8 principals -10 teachers -8 food preparers -1 local site coordinator -1 Ontario Fruit and Vegetable Growers' Association (OFVGA) representative	Schools
**Carbone et al. (2016)** [27]	The particular Farm to Preschool project identifies three parts: 1) activities to establish a system through which preschools can purchase local produce at a lower cost 2) activities to ensure children, staff and families know how to prepare, serve healthy foods, and consume healthy foods 3) activities introducing changes to the early education regulations aiming toward higher nutrition standards, and purchase of locally grown foods. Some of the services offered are: trainings, purchases of products, family field trips, mobile markets and a healthy snack grant. (p.179)	USA (Springfield, Maryland)	classroom observations interviews administrator surveys	(approximately) 44 students observed (age 3–5) (4 observation sessions -one prior to evaluation, 3 during evaluation; estimated that on average 11 students observed per classroom) 4 food service staff members 4 teachers 5 administrators	Preschools
**Chen et al. (2014)** [29]	The intervention consists of three parts: 1) recipe development: development of seven food recipes from Hmong, Latino, mainstream American culture with the help of students and parents participating in the intervention 2) classroom component: 20 min in-class monthly tasting activities from February—May 2012, implemented by nutrition educators and teachers 3) family component: after each in-class demonstration and tasting activity, children were given 'food kits' containing recipes and ingredients as well as preparation tools to take home, so as to prepare the meal with their parents. (p.115–116)	USA (Northern California)	focus groups	28 parents	Schools
**Cirillo et al. (2018)** [34]	Although the Farm to School programs included in the study varied, they all entailed the following parts 1) nutrition education in the classroom 2) improvements to the food options in the cafeteria 3) engagement with the local community. One of the most common mentioned actions of the programs was a school garden, and the use of the fruits and vegetables from the garden, for consumption by the children. (p.3)	USA (Vermont)	semi-structured interviews	10 principals	Schools
**He et al. (2012)** [25]	The Norther fruit and vegetable program entails distribution of free fruit and vegetables snacks. Fruit and vegetables were distributed three times per week. The program was piloted in December 2006 to May 2007 and expanded and implemented in 2007/2008 school year. (p. 592)	Canada (Ontario)	focus groups	139 students	Schools
**Hector et al. (2017)** [18]	The intervention provides supplementary, free fruits and vegetables to schools that participate in the Crunch and Sip initiative. The Crunch and Sip initiative encourages teachers to incorporate a 'snack' break during their usual classes, and eat fruits and vegetables together with the children. Although as part of Crunch and Sip, children are asked to bring fruits and vegetables from home, the current intervention supplements free fruits and vegetables for children who have not brought such a snack from home. The intervention was implemented for 10 weeks, in 2014, and consisted of the distribution of bananas, apples and carrots. (p. 239)	Australia (Western Sydney)	questionnaires	55 teachers 4 key contacts in participating schools	Schools
**Jørgensen et al. (2014)** [26, 36]	The Boost intervention combined educational and environmental strategies within the school, home and community, such as: curriculum activities, daily free fruit and vegetables at school, parental newsletters and fact sheets for sports and youth clubs. The intervention lasted for 9 months (September 2010-May 2011). (p. e2)	Denmark	focus groups individual interviews	22 teachers	Schools
**Knapp et al. (2019)** [28]	A school-based kitchen garden program that consists of a core curriculum, taught by specialized teachers, during school hours, which entails the involvement of students in growing, harvesting, preparing and eating the food from school gardens. In addition, the program aims for family and community involvement through: Family Food Nights, Open Garden Days, and Parent Cooking Classes. (p. 669)	USA (New Orleans, Louisiana)	focus groups	27 students 17 parents 17 teachers	Schools
**Lee et al. (2019)** [35]	The focus of the study is on farm to school programs generally, which are described as involving a range of activities linked to the promotion, procurement, serving of local food—fruits and vegetables and teaching related to nutrition and local food production. (p. 374)	USA (Ohio)	semi structured interviews focus groups	194 practitioners and community residents 18 experts	Community
**Lin et al. (2016)** [17]	The US Department of Agriculture Fresh Fruit and Vegetable Program, is a federally funded program, where schools are given funds to purchase fresh fruits and vegetables for children, prepare and serve them as snacks, outside of the regular school meals. The program was expanded to all US states in 2008, while in 2010 it was limited to elementary schools only. (p. 321)	USA (Indiana)	questionnaires (open ended item for program comments)	3811 students	Schools
**Potter et al. (2011)** [31]	The Mississippi Fruit and Vegetable Pilot Program consists of distributing free fruits and vegetables to children, as snacks during the school day. The pilot program was implemented in 2004/2005 school year. (p. 203)	USA (Mississippi)	interviews focus groups	11 program staff 6 administrators 42 students 19 parents	Schools

**Table 2:** Characteristics of primary studies by target group

**Author, year**	**Data collection method**	**Sample size and target group**	**Students/children**	**School based implementing actors (Teachers/Principals/Cafeteria managers/food preparers/on site coordinators/program staff/administrators)**	**Parents**	**Suppliers/producers/distributors**	**Other (practitioners, community residents, experts)**
**Aarestrup et al. (2014) **[26, 36]	focus group interviews class observations telephone interviews	6 schools class observations—no estimate of number of persons observed 111 students (13 years old) 13 teachers 18 suppliers	111	13		18	
Bateman et al. (2014) [33]	phone interviews	10 producers (farmers) 5 distributors				15	
**Bogart et al. (2018) **[32]	interviews focus groups	16 teachers 16 principals 14 cafeteria managers 154 students	154	46			
**Bouck et al. (2011) **[30]	qualitative interviews	28 stakeholders: -8 principals -10 teachers -8 food preparers -1 local site coordinator -1 Ontario Fruit and Vegetable Growers' Association (OFVGA) representative		27		1	
**Carbone et al. (2016)** [27]	classroom observations interviews administrator surveys	(approximately) 44 students observed (age 3–5) (4 observation sessions -one prior to evaluation, 3 during evaluation; estimated that on average 11 students observed per classroom) 4 food service staff members 4 teachers 5 administrators		13			
**Chen et al. (2014)** [29]	focus groups	28 parents			28		
**Cirillo et al. (2018)** [34]	semi-structured interviews	10 principals		10			
**He et al. (2012)** [25]	focus groups	139 students	139				
**Hector et al. (2017)** [18]	questionnaires	55 teachers 4 key contacts in participating schools					
**Jørgensen et al. (2014)** [26, 36]	focus groups individual interviews	22 teachers		22			
**Knapp et al. (2019)** [28]	focus groups	27 students 17 parents 17 teachers	27	17	17		
**Lee et al. (2019)** [35]	semi structured interviews focus groups	194 practitioners and community residents 18 experts					212
**Lin et al. (2016)** [17]	questionnaires (open ended item for program comments)	3811 students					
**Potter et al. (2011)** [31]	interviews focus groups	11 program staff 6 administrators 42 students 19 parents	42	17	19		
**TOTAL**			473	165	64	34	212

### Quality assessment results

The quality assessment was done independently by two authors (B.M. and M.S.). As all papers were evaluated positively on at least seven (13 of the 14 papers on at least nine) checklist points, the overall conclusion that all 14 articles were of sufficient methodological quality to be included in the review was made through consensus of the two authors. Some general remarks can be made based on the assessment. Papers were found to be especially weak in regard to ‘reflexivity’ and ‘theoretical framework’. In particular, both authors agreed that only two out of the 14 papers had a theoretical frame of reference. Namely, Jørgensen et al. [26] used the Diffusion of Innovations Theory [37] in the design of the study, while Bogart et al. [32], used the RE-AIM framework in the design of the study and analysis of the results. For full overview of the quality assessment results by the two authors (B.M. and M.S.) as well as notes on the discussion following the assessment, and overall evaluation, please refer to Additional files 3 and 4.

### Framework analysis: Consolidated Framework for Implementation Research

**Fig. 2:**
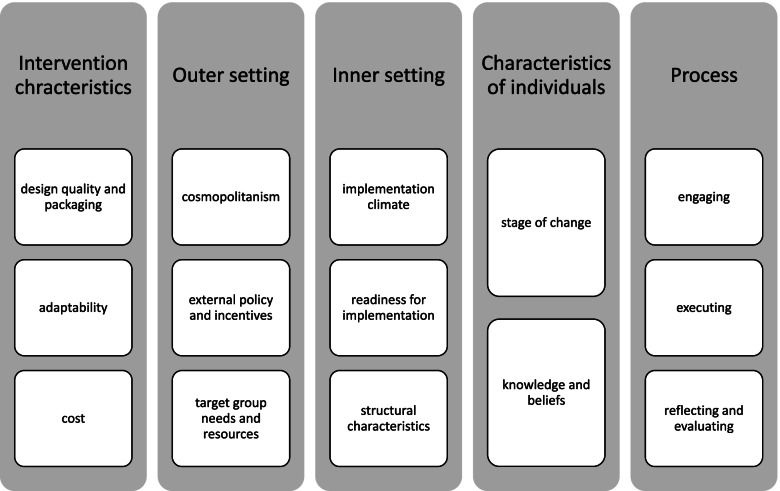
Overview of main findings

Figure 2 gives a visual overview of the main findings, across the five domains of CFIR. The constructs listed under the appropriate domain are those that were found to be most widely present (in a minimum of 5 papers) across the 14 papers included in this review.

Additional file 5 provides an overview of the text extracted from all 14 papers as it is coded under each domain, construct and sub-construct of CFIR, with the color code reflecting the intensity of coding under each construct, from each paper (red signifying that 5 or more text segments covering different topics which would fall under the construct have been coded). In the following the results will be presented by the five domains of CFIR.

Additional file 6 provides examples of text segments as coded under each domain and construct. 

#### Intervention Characteristics

The construct most widely addressed across papers and coded with greatest intensity overall as well as within the *intervention characteristics* domain, was ‘**design quality and packaging**’. Different aspects of this construct were discussed in 13 out of the 14 papers. Studies emphasized the importance of the quality of the fruit and vegetables provided [25, 30, 33, 36] their taste [25] and texture [27]. Vegetables were less desired [17] and dips or seasoning were seen as a way to make them more attractive to children [25, 27, 31]. Some studies emphasized preference for certain types of fruit and vegetables such as bananas, pineapples and carrots [18, 27] while another study found that ‘exotic’ fruits (non-local) were preferred [31]. The manner in which the fruit and vegetables were packaged and presented to children was also highlighted as important [31, 36]. For instance, in Aarestrup and colleagues, fruit and vegetables were cut during breaks prior to a lesson, while children were allowed to eat the fruit and vegetables after the lesson, a process that caused browning of the produce, and thus, child reluctance to eat them [36]. Studies also emphasized that a greater variety [17, 25, 30, 31, 36] and frequency [17, 25] in the fruit and vegetables provided was often lacking. The quantity of fruit and vegetables provided however was by some studies found to produce waste [30, 36]. Finally, one study found that number and type of activities aimed at parents and community members were insufficient [28].

Two other constructs within this domain which were also widely present across papers were ‘adaptability’ and ‘cost’. In regard to ‘**adaptability’**, the findings based on the Boost intervention are particularly worth highlighting [36]. The intervention was designed in such a way as to leave the decision of when to have a fruit and vegetables break up to the teachers, thus providing often necessary flexibility. However, the process evaluation found that the decisions some teachers made about the timing of the fruit and vegetables break were contrary to the desires of the children, resulting in the browning of the produce, and reluctance to consume the same. ‘**Cost’** was a relevant determinant in two particular respects. Studies emphasized the importance of fruit and vegetables which are free, as that was found to be helpful for children of lower socio-economic background in particular [25, 31]. In cases where schools had to seek funds to finance the intervention, the instability of finances was seen as a barrier [27, 35] this was specifically the case with some farm to school programs [35]. Finally, a consideration for distributors and producers was the possibility of making profit, should they take part in such interventions [33].

The perspective of suppliers is worth highlighting as evident under the construct of ‘**complexity’**, which overall was not widely addressed across papers compared to ‘design quality and packaging’, ‘adaptability’ and ‘cost’. Some interventions required specific ways of packaging the fruit and vegetables, which for some suppliers meant more effort in order to comply [36]. Overall, in regard to ‘complexity’, studies highlighted the overall duration of interventions, and the time investment demanded to implement the different components, by teachers in particular [26, 32].

#### Outer setting

The most widely present constructs from the *outer setting* were ‘cosmopolitanism’ (8 out of 14 papers) and ‘external policy and incentives’ (7 out of 14 papers). When reporting on ‘**cosmopolitanism’**, the most commonly found barriers and facilitators were linked to the relationship between the school and the farm/producers/suppliers/delivery service [18, 30, 33, 34, 36]. Studies found the lack of communication and misunderstandings on delivery times to be a significant barrier, in particular at the start of an intervention [30, 33, 36]. A developed relationship between farms and schools however, was found to be a facilitator to implementation [34]. Finally, one study reported on cooperation between schools in sharing available storage space for the fruit and vegetables [31]. ‘**External policy and incentives**’ was another *outer setting* construct often addressed in papers. Whereas the availability of external funding was found to be a facilitator to implementation for schools [27, 30, 35] limited external funds were a barrier [32]. Studies also found that consistency between the intervention and food related guidelines coming from the municipal or national level were a facilitator to implementation [18, 32] however, support of community leaders was important when the intervention was not in compliance with food regulations already in place [35].

Text linked to the construct ‘**patient needs and resources’** (in the context of this study ‘patient’ refers to the primary target group-children) was present in 5 of the 14 papers reviewed. When the intervention was perceived to address the needs of children in regard to their overall health – mental and physical, it facilitated the implementation [34]. However, the content of this construct, primarily pointed to barriers of implementation. For instance, some social dynamics amongst teenagers, as well as gender based differences in reactions to the intervention were somewhat overlooked in the design and implementation of the Boost intervention [36]. Another study found that having children distribute some of the gifts of the intervention (such as promotional bookmarks) to their peers made them feel uncomfortable, and that the educational measure as part of the intervention was too difficult for children to comprehend [32].

Finally, ‘**peer pressure’** was found to be a relevant construct in only one of the included studies, and it is worth noting, as it primarily represents the views of distributors [33]. Namely, some distributors took part in the intervention because it recognized the importance given to local produce by the community overall, and offering their services was thus perceived to give them an advantage over their competitors [33].

#### Inner Setting

Highly prevalent constructs of the *inner setting* were found to be ‘implementation climate’ (9 out of 14 papers) and ‘readiness for implementation’ (8 out of 14 papers). Within the ‘**implementation climate’** construct, the sub-construct most often addressed was ***compatibility*** [18, 26, 31, 32] of the intervention, mostly with the workload of teachers but also with the educational curriculum and other ongoing programs [18, 35, 36] as well as with food related guidelines [27, 35]. Text linked to the sub-construct ***relative priority*** was often a barrier, as teachers had a tendency to prioritize other, often curriculum related obligations [32, 36]. Under the sub-construct ***organizational incentives and rewards***, one study emphasized the importance of celebrating the overall success of the children, due to the intervention, as such a celebration was found to be a strong motivating factor for teachers [34]. The same study identified the offering of symbolic fees to those implementing the intervention as a facilitator, in order to recognize their time and effort [34]. Another study addressed the same sub-construct from the perspective of suppliers, noting that the chance for branding, as well as the possibility to support what was perceived as a good cause was an incentive for suppliers to take part in the intervention, although profits may not have been as enticing [36]. Finally, results linked to the sub-construct ***goals and feedback***, emphasized the importance for teachers to clearly understand the objectives of the intervention [26].

Text linked to the construct **‘readiness for implementation’**, mostly belonged to the sub-construct ***available resources*** [26, 27, 30–32, 34–36]. Findings emphasize the importance of trainings, workshops, materials provided as well as hiring additional support staff for implementation of the intervention in the school setting, as important facilitators, which could be perceived as barriers when materials were lacking due to delayed delivery, or no additional staff could be hired due to budget restrictions. However, within this sub-construct, the most commonly mentioned resource was time, primarily serving as an important barrier for teachers, in implementing the intervention. The lack of functionality of a website linked to the intervention was identified as a barrier as part of the ***access to knowledge and information*** sub-construct [32]. Finally, the role of the principal of the school was found relevant under ***leadership engagement***, serving as a motivating factor for teachers and their own commitment to intervention implementation [26, 32].

Two additional constructs are also worth noting within the *inner setting*, which although not as prevalent as ‘implementation climate’ and ‘readiness for implementation’, were nonetheless present – ‘structural characteristics’ (6 out of 14 papers) and ‘networks and communications’ (4 out of 14 papers). Under ‘**structural characteristics’**, findings emphasized the importance of storage space, kitchen space, having containers, utensils and refrigerators but also the location of the school as important, as more distant schools were a challenge for distributors to reach [27, 28, 30, 31, 33, 36]. Strong ‘**networks and communication’**, in particular among teachers, administrators and kitchen/food service staff within the school was also identified as important across studies [27, 28, 32, 34].

#### Characteristics of Individuals

Among the constructs related to the *characteristics of individuals* domain, text pertaining to ‘stage of change’ (9 out of 14) and ‘knowledge and beliefs about the intervention’ (8 out of 14) were most present among the included papers. Text linked to ‘**stages of change’** was common, but superficial, primarily emphasizing the importance of staff ‘buy in’ as a facilitator and important for success of the intervention [27]. Educating teachers about the intervention and related to that, workshops were identified as methods that could ensure teacher ownership of the intervention, and thus its sustainability [26, 34]. Expression of enthusiasm by teachers toward the opportunity to teach in an applied way was also identified as a facilitator and thus, an indication of the individual stage of change of those individuals [28]. Distributors in one study saw their participation in the intervention as their moral obligation and thus were committed to providing fresh, local produce to their school and through it to their community [33].

Perceptions of behavior change were expressed under ‘**knowledge and beliefs about the intervention’**, from the perspective of persons involved in the implementation of the intervention, most commonly teachers [18, 28, 30, 31, 33, 34]. Teachers reported the perception that the intervention contributed to children eating healthier [18, 30, 33] improvement of child physical and cognitive health [18, 31] development of child life skills and improvement of relationships among children and school staff [34]. Only one study reported that teachers expressed doubts in the expected impact of the intervention, which was increase in fruit and vegetables consumption among children [26].

Two papers had content in regard to the construct ‘**individual identification with the organization’** [26, 33]. Teachers taking part in the Boost intervention expressed their feeling of responsibility to implement the intervention, after their school had committed to participate [26] while producers and distributors taking part in a farm to school program expressed their dedication to respecting the contract signed with the school [33]. Finally, ‘**self-efficacy’** related text was indicated by teachers taking part in the Boost intervention, as they found teaching unfamiliar topics was a familiar task, and thus facilitating implementation, while their ability to control the classroom even when food fights occurred also showed to be important for implementation [26, 36].

#### Process

The dominant construct of the *process* domain was ‘engagement’ (12 out of 14), followed by ‘executing’ (6 out of 14), ‘reflecting and evaluating’ (6 out of 14) and finally ‘planning’ (4 out of 14). Within ‘**engaging’** papers referred most often to the sub-construct of ***external change agents***, in particular, parents [27–29, 31, 32, 35] farmers [34, 35] community leaders [35] and college teachers [26]. For instance, the lack of awareness by community leaders in regard to the intervention was identified as a barrier to implementation [35]. However, the role of parents as external change agents was twofold, both as facilitators in ensuring the intervention benefits their children [28] but also as a secondary target group which could potentially improve their own eating practices, as well as those of the family as a whole [29]. One study identified parental buy in as a key facilitator [27], while another study identified the lack of parental support as a key barrier [35]. Further, two papers [28, 29] mentioned components which actively involved parents. A third [32] described a take home activity that aimed at influencing what families bought and ate at home, as children shared their knowledge with their parents as to what is and is not considered healthy food. In addition, under the construct of ‘engaging’, school teachers and the school board were identified as ***opinion leaders*** [27, 30, 34] in particular teachers as role models was seen as an important facilitator. The appointment of intervention coordinators, as ***internal implementation leaders*** was another facilitator [26]. Finally, several studies emphasized the importance of a ***champion*** for the success of implementation, identified to be someone from the school staff or an industry contact [18, 35].

Within the construct of ‘**executing’**, the most common barrier identified was the delivery time, or altogether lack of delivery of the fruit and vegetables [30, 31, 36]. In addition, papers stressed the importance of having a method of distribution of the fruit and vegetables, once inside the school [18, 31, 35]. Finally, unexpected food games with the fruit and vegetables in the process of executing the intervention were identified as a barrier [36].

In regard to **‘planning’**, studies reported having a planning committee [34] a schedule as a visual tool [26] and a back-up plan in case there are delivery problems as important [31]. Examples of back-up plans were serving more than the planned quantity of fruit and vegetables in situations when there is danger they may brown, or serving dry fruit when delivery did not occur [31].

Finally, under the construct of ‘**reflecting and evaluating’**, studies reported on formal evaluation results being fed back into the implementation of the intervention, [26, 36] but also on more informal learning and reflecting processes which were then again facilitating the implementation [31, 34].

#### Themes not within CFIR

Through our review, we have come across texts which could not be coded under the current CFIR constructs, referring to one dominant theme – **children’s perceptions of value of the intervention** and **perception of personal behavior change due to the intervention**. Although perceptions of behavior change from the perspective of teachers were presented as part of the ‘knowledge and beliefs’ construct, within the *characteristics of individuals* domain, the determination was made that this domain contains views of implementers rather than the target group. Thus, target group perceptions of behavior change could not be coded. Nonetheless, how the target group perceived changes to their behavior due to the intervention, in addition to their views on the content of the intervention (which is part of ‘design quality and packaging’), was highlighted as an important determinant of implementation by several papers, and thus, must be included [25, 28, 29, 31, 36]. The following extractions from several of the noted studies, give an example of the text:

*Participants perceptions of the free fruit and vegetable snacks: increased fruit and vegetable intake, tried new fruits and vegetables, changed fruit and vegetable preferences* [25]

*Students noted that the snacks helped prevent hunger if they skipped a meal or had lunch later in the day (…) appreciated the program because they felt it demonstrated that school staff cared about them *[31]

*The pupils appreciated that the fruit and vegetable programme was for everyone and some pupils expressed that it became a habit to eat fruit and vegetable in class and that they affected each other’s eating habits *[36]

We have taken the presented text reflecting perceptions of children as the primary target group as a facilitator (when children express positive perceptions) or barrier (when children express negative perceptions) to implementation.

## Discussion

This review highlights the importance of the following CFIR constructs, as determinants in the implementation of fruit and vegetables interventions in schools: 1) *intervention characteristics* domain: ‘design quality and packaging’, ‘adaptability’ and ‘cost’; 2) *outer setting:* ‘cosmopolitanism’, external policy and incentives’ and ‘patients’ needs and resources’; 3) *inner setting:* ‘implementation climate’, ‘readiness for implementation’ and ‘structural characteristics’; 4) *characteristics of individuals*: ‘individual stage of change’, ‘knowledge and beliefs about the intervention’ and finally of 5) *process:* ‘engaging’, ‘executing’ and ‘reflecting and evaluating’. The review stresses the dual role of parents as both supporting the implementation and targets of the intervention. Positive child perceptions of the value of the intervention and perceived behavior change due to the intervention were reported as relevant facilitators to implementation across several studies.

### Intervention Characteristics

The importance of **quality** and **variety** of the fruit and vegetables is consistent with research looking at school level factors that may impact fruit and vegetables consumption in middle and high schools, where quality of fruit was significantly associated with a 44% increase in fruit consumption, and variety of vegetables in the form of salad bars with a 48% increase in vegetable consumption [38]. Consistent with recent findings on barriers and enablers of implementation of the Norwegian school meal guidelines [39], **adaptability** in the context of our review was also found to be both a facilitator and barrier to implementation.

### Inner setting

Within the *inner setting domain,* the sub-constructs of ***compatibility, relative priority*** (**implementation climate**) and ***available resources (readiness for implementation)***, all point to the importance of teacher workload in regard to regular curriculum activities, and time pressures that teachers face when implementing fruit and vegetables interventions. This is consistent with findings on implementation of nutrition policies in schools generally, where ***training support and resources*** are found to be key facilitators while ***competing priorities*** and ***time consuming nature*** of implementing nutrition policies in schools are barriers [40]. The importance of time as a barrier to implementation is also emphasized in the review by Ismail and colleagues (2021), focusing on interventions providing fruit and vegetables as snacks [14], as well as by Swindle and colleagues (2019) who find time constraints as a barrier to implementing a nutrition intervention in a child care setting [41].

### Process

In the context of our review, parents, as ***external change agents*** were found to have a dual role as supporters of implementation, but also secondary targets of the intervention. Consistent with our findings, Ismail and colleagues also highlight the importance of parental engagement for the success of school based dietary interventions [14]. Literature more widely recognizes the significant role of parents in shaping family, and thus, child eating practices [42–44].

#### CFIR: application

One of the advantages of using CFIR for synthesis of results is that it enables comparison of findings across studies using the same framework [13]. A recent upcoming meta-review looks at determinants of implementation of healthy diet, physical education, and sedentary behavior policies finding strong support for ‘cost’, ‘cosmopolitanism’, ‘external policy and incentives’, ‘implementation climate’, ‘readiness for implementation’ and ‘knowledge and beliefs about the intervention’ across different settings, and ‘patients needs and resources’, ‘structural characteristics’ and ‘engaging’ specific to school settings [45]. The findings of our review differ notably in two specific areas: first, in identifying the constructs ‘design quality and packaging’ as part of the *intervention characteristics*, and the construct ‘executing’ as part of the *process*; and, second, in lacking the prevalence of ‘complexity’ highlighted in Lobczowska and colleagues [45]. The reasons for the differences may be that in the current review we included interventions broadly (as defined in introduction), rather than policies only, and in particular, direct provision of fruit and vegetables, which may explain the emphasis on the quality of the produce as part of the design quality and packaging construct.

#### CFIR: shortcomings and space for improvement

However, CFIR is not without its shortcomings. It’s one-dimensionality, not only prevents a distinction of micro, meso and macro level factors, which other frameworks do offer [46, 47] but as Lobczowska and colleagues [45], also highlight, it does not distinguish between views and perceptions of the target group, delivery system actors (responsible for implementation) and support system actors (responsible for providing support for implementation) [48]. Flottorp and colleagues (2013) compiled a checklist of factors that influence healthcare practice, where ‘patient factors’ is one of the domains, containing constructs such as patient knowledge, attitudes and motivations for behaviour change [49]. As research in the field of implementation science is moving forward, the importance of recognizing different groups as part of implementation, with differing perceptions, knowledge and beliefs, that may serve as determinants in their own right, is at the forefront [48, 50, 51].

In the context of the current review, the authors did make the decision that constructs under the *characteristics of individuals* domain will reflect the perspectives of implementing actors only. The decision to do so was made after a thorough exploration of the domain and its constructs [24] and a subsequent discussion among the authors (B.M and N.L). It was the consensus that the offered descriptions of the constructs under *characteristics of individuals* domain would most adequately reflect the views and perceptions of implementers rather than the target group. The same process was followed when deciding to place the perceptions and views of parents in regard to the intervention, as well as parental behaviour change due to the intervention under the construct ‘engaging’. However, these were our choices, and a more thorough revision of CFIR may be needed to reflect the progress made in implementation science more widely.

Our decision to make a distinction between the views expressed by different actors relevant to implementation, also led us to introduce a new theme coded outside of CFIR, containing text that reflects the *perceptions of value of the intervention and perceptions of behavior change due to intervention by children* as the primary target group*.* To the best of our knowledge, there is little input from literature up to date, emphasizing the importance of perceptions of value of an intervention, or perceptions of behavior change, by the target group, as a determinant of implementation. Although, the importance of children liking the fruit and vegetables intervention has been associated with the effect of the intervention reported on in both the Pro Children and PRO GREENS studies [52, 53].

Concepts close to the newly identified theme may be ‘observability’ and ‘attitude’, both of which could potentially link to ‘acceptability’. The concept of ‘observability’ [54–56] is defined as ‘the degree to which the results of an innovation are visible to others’ [54], while attitude [57–59] is the ‘degree to which a person has a favorable or unfavorable evaluation or appraisal of the behavior in question’ [58]. Attitude is partially shaped by beliefs about a behavior [58, 60] where ‘we favor behaviors we believe have largely desirable consequences’ [58]. A study looking at barriers and facilitators to integrating innovations in hospital settings, identified attitudes toward the innovation, partially shaped by perceived benefits of the innovation to patients, as a facilitator, however, expressed from the perspective of implementers rather than the target group [61]. Acceptability as the perception that an intervention is ‘agreeable, palatable, or satisfactory’ [62] is sometimes used in existing research to express views of the target group [63]. However, the construct, although widely present in implementation science research, is primarily conceptualized as an implementation outcome rather than a determinant [62] and seems too narrow to include *perceptions of value* and *perceptions of behavior change*. Further, neither ‘observability’, ‘attitude’ nor acceptability as such are included in CFIR. Thus, based on the current research, we would recommend a definition of ‘acceptability’ as an implementation determinant, which would be inclusive of concepts such as ‘observability’ and ‘attitudes’. This would be the closest we could identify to reflect our theme – *perceptions of value of the intervention*, and *perceptions of behavior change due to the intervention*. We would further recommend that any or all aforementioned concepts are defined in such a away, as to allow space for reflecting and clearly distinguishing the views of the target group, from those of implementing actors.

Changes to CFIR in this direction may be upcoming, with latest (checked October 2021) references found in CFIR website [24] to a version 2 of the framework, that would contain two new sub-constructs under ‘**engaging’**: ***key stakeholders*** and ***innovation participants***. However, further consideration of actor roles, and specification of these constructs (with due consideration to concepts such as ‘observability’, ‘attitude’ and ‘acceptability’) are needed to guide reserachers when using the framework.

### Limitations, strengths and recommendations

This paper has explored the determinants of implementation based on CFIR, that would be relevant to interventions providing fruit and vegetables in schools, as well as recommend possible amendments to the framework when applied to school settings, and in regard to direct provision interventions. However, there are several limitations to this research. Few of the included studies used an implementation framework to analyze their findings, thus the determination to place extracted text under each of the CFIR constructs was made by the authors, based on what the text segments implied as identified barriers or facilitators to implementation. CFIR poses the additional challenge, that it allows for double coding of text under its constructs. Although double coding was avoided by the authors, and decisions on placing text under each construct was performed on basis of consensus of at least two authors, other researchers may code the text differently. Finally, although CFIR does not make a distinction between target groups, it was our own decision to do so in the context of this work. The potential shortcomings may also be seen as its strengths. We have used a widely applied determinant framework from implementation science to synthesize results that now further offers the opportunity to compare findings on barriers and facilitators to implementation across studies from different fields. In doing so, we have highlighted which may be the most relevant constructs when looking at school based, direct provision of fruit and vegetables interventions, thus giving other researchers the opportunity to be more selective when conducting their own implementation evaluations of similar future interventions, in similar settings. Finally, we have identified ways in which CFIR can be revised and expanded, so as to keep up with the progress made in implementation science and be applicable to a variety of settings.

Based on our findings, we propose the following recommendations:

### General

- Use Fig. 2 as a guide as to constructs that should be explored when investigating barriers and facilitators to implementation of food related interventions in kindergartens and schools. Of those highlighted, less commonly touched upon constructs across the papers (such as cost, target group needs and resources, structural characteristics, executing, reflecting and evaluating) may be in need of further exploration.
- Consider the needs of different target groups during intervention design: children and parents
- Explore acceptability of the intervention by all actors involved (in the implementation and as target groups): teachers, school administrators, children, parents

### Particular to interventions which provide fruit and vegetables to children in kindergartens and schools

- Explore what is possible and acceptable in regard to ‘design quality and packaging’ with teachers, target group(s) and those delivering the fruit and vegetables
- Considering the importance of ‘cosmopolitanism’ the relationship between those delivering the fruit and vegetables and those receiving it at the kindergarten/school deserves particular attention. Time and effort should be dedicated to establishing these relationships from the very beginning, and those relationships should be nurtured throughout implementation.

## Conclusion

We concluded that CFIR offers a systematic way to identify and organize barriers and facilitators to implementation of interventions in the kindergarten and school setting. Further, we have singled out the most relevant constructs (Fig. 2) which identify barriers and facilitators for interventions that provide fruit and vegetables to children in kindergartens and schools. However, revisions are encouraged to allow adequate space for perceptions of various implementation actors and the target group.

## Supplementary Information

**Additional file 1.** Documentation of literature search and data extraction and analysis

**Additional file 2.** Definitions and Coding Clarifications

**Additional file 3.** Quality assessment

**Additional file 4.** Quality assessment process notes

**Additional file 5.** Grid Overview table

**Additional file 6. **Grid example

## Data Availability

The datasets used and/or analyzed during the current study are available from the corresponding author on reasonable request.

## Abbreviation

CFIR: Consolidated Framework for Implementation Research
