# Supplementary material for: Barriers and facilitators to implementation of direct fruit and vegetables provision interventions in kindergartens and schools: a qualitative systematic review applying the consolidated framework for implementation research (CFIR)
Source: Int J Behav Nutr Phys Act. 2022 Jan 31;19:11. doi: 10.1186/s12966-022-01246-8 (PMC8805256; doi:10.1186/s12966-022-01246-8)
Supplement: Supplementary file 1 — Additional file 1. Documentation of literature search and data extraction and analysis [file 12966_2022_1246_MOESM1_ESM.docx]

Documentation of literature search

Documentation on the literature search for:

1. What is the impact of school fruit and vegetable interventions?
2. How are fruit and vegetable interventions implemented in kindergartens, primary and secondary schools?

The following databases were searched:

| **Database** | **Number of retrieved references** |
| --- | --- |
| MEDLINE (Ovid): | 2460 |
| Embase (Ovid): | 3656 |
| PsycINFO (Ovid): | 986 |
| ERIC (Ovid) | 951 |
| Cochrane Library Reviews: | 4 |
| Cochrane Library Trials | 683 |
| Number of references before deduplication: | 8740 |
| Number of references after deduplication: | 5240 |

All searches were done 8 November 2019 by Hilde Strømme, Adviser at the Medical library, University of Oslo.

Number of hours spent: 15,5

**Ovid MEDLINE(R) ALL <1946 to November 07, 2019>**Date searched: 8 November 2019
Number of hits: 2460

| 1 | Fruit/ or Vegetables/ or (fruit* or vegetable*).tw,kw,kf. | 147089 |
| --- | --- | --- |
| 2 | Schools/ or Schools, Nursery/ or (school* or kindergarten*).tw,kw,kf. | 281291 |
| 3 | exp Policy/ or exp Health Promotion/ or Program Evaluation/ or Implementation Science/ or (intervention* or scheme* or policy or policies or promoti* or promote* or program* or implement* or "process evaluation").tw,kw,kf. | 3140917 |
| 4 | and/1-3 | 2460 |

**Embase Classic+Embase 1947 to 2019 November 07**Date searched: 8 November 2019
Number of hits: 3656

| 1 | exp fruit/ or exp vegetable/ or (fruit* or vegetable*).tw,kw. | 365740 |
| --- | --- | --- |
| 2 | school/ or high school/ or kindergarten/ or middle school/ or nursery school/ or primary school/ or (school* or kindergarten*).tw,kw. | 374786 |
| 3 | intervention study/ or exp policy/ or exp health promotion/ or exp program evaluation/ or (intervention* or scheme* or policy or policies or promoti* or promote* or program* or implement* or "process evaluation").tw,kw. | 4175830 |
| 4 | and/1-3 | 3656 |

**PsycINFO 1806 to October Week 4 2019**

Date searched: 8 November 2019
Number of hits: 986

| 1 | (fruit* or vegetable*).tw. | 19066 |
| --- | --- | --- |
| 2 | Schools/ or Elementary Schools/ or High Schools/ or Junior High Schools/ or Kindergartens/ or Middle Schools/ or Nursery Schools/ or (school* or kindergarten*).tw. | 405001 |
| 3 | School Based Intervention/ or Health Promotion/ or Program Evaluation/ or (intervention* or scheme* or policy or policies or promoti* or promote* or program* or implement* or "process evaluation").tw. | 997245 |
| 4 | and/1-3 | 986 |

**ERIC <1965 to August 2019>**

Date searched: 8 November 2019
Number of hits: 951

| 1 | (fruit* or vegetable*).tw. | 3960 |
| --- | --- | --- |
| 2 | Schools/ or Day Schools/ or Elementary Schools/ or Middle Schools/ or Private Schools/ or Public Schools/ or Rural Schools/ or Secondary Schools/ or Suburban Schools/ or Urban Schools/ or Kindergarten/ or (school* or kindergarten*).tw. | 673811 |
| 3 | exp Intervention/ or exp policy/ or Health Promotion/ or Health Programs/ or Program Implementation/ or (intervention* or scheme* or policy or policies or promoti* or promote* or program* or implement* or "process evaluation").tw. | 776545 |
| 4 | and/1-3 | 951 |

**The Cochrane Library**

Date searched: 8 November 2019
Number of hits: 4 Reviews, 683 Trials

| #1 | MeSH descriptor: [Fruit] this term only | 1551 |
| --- | --- | --- |
| #2 | MeSH descriptor: [Vegetables] this term only | 1214 |
| #3 | (fruit* OR vegetable*):ti,ab,kw | 8474 |
| #4 | #1 OR #2 OR #3 | 8474 |
| #5 | MeSH descriptor: [Schools] this term only | 1815 |
| #6 | MeSH descriptor: [Schools, Nursery] explode all trees | 37 |
| #7 | (school* OR kindergarten*):ti,ab,kw | 31886 |
| #8 | #5 OR #6 OR #7 | 31886 |
| #9 | MeSH descriptor: [Policy] explode all trees | 731 |
| #10 | MeSH descriptor: [Health Promotion] explode all trees | 5996 |
| #11 | MeSH descriptor: [Program Evaluation] this term only | 5824 |
| #12 | MeSH descriptor: [Implementation Science] this term only | 15 |
| #13 | (intervention* OR scheme* OR policy OR policies OR promoti* OR promote* OR program* OR implement* OR "process evaluation"):ti,ab,kw | 442795 |
| #14 | #9 OR #10 OR #11 OR #12 OR #13 | 442837 |
| #15 | #4 and #8 and #14 | 687 |
| #16 | #15 in Cochrane Reviews, Cochrane Protocols | 4 |
| #17 | #15 in Trials | 683 |

**Implementation Science Journal**

Date searched: 6 July 2020
Number of hits: 156

| #1 | Fruit | 57 |
| --- | --- | --- |
| #2 | Vegetable | 48 |
| #3 | fruit, vegetable | 51 |

Search of Implementation Science was conducted by author Biljana Meshkovska.

**Data extraction and analysis: step by step process**

The 14 articles were divided among three authors (D.A.S, J.M.S. and J.W), who carefully read through the studies, identifying any ‘text segments’ which could independently stand as results (second order concepts), following from the analysis of primary data in the selected papers. These text segments were then extracted in excel sheets where the rows listed each of the included papers, while the extracted segments were entered vertically, so that each column represented the totality of the results from the particular study. This process was repeated for all 14 articles by the first author (B.M.) and each extracted text segment was thus verified. Where necessary, text segments were added, supplemented or expanded to provide more context. Although direct quotations from target groups were included, together with text segments in some cases, this was done to provide for context of the findings of the primary studies, rather than to include the quotations for the purpose of analysis of primary results (first order analysis) in this systematic review. As recommended by Malterud (2019), results were extracted from the entirety of the content of the papers included (abstract, introduction, results/findings, discussion, conclusion) [1]. Literature references made in the primary studies, that may have been intertwined with extracted results, were excluded and only the results were taken into consideration.

Initial coding was done by one author B.M., and verified by the principal investigator N.L. Any conflicts were resolved through discussion and consensus, and the discussion and final decisions have been recorded in detail. The final analysis was conducted by the two authors (B.M. and N.L.). Each primary study was considered as a ‘case’ and consequently, the results of each study now extracted as text segments which could independently stand, were deductively coded within the domains and constructs (as well as sub-constructs) of CFIR. Where text segments were not explicitly identified as determinants: ‘barriers’, ‘facilitators’ or both, in the context of the primary study, this determination was made by the authors (B.M. and N.L.). In these cases, the text segment was understood as an ‘assumed’ or ‘implied’ determinant. Text segments were coded under each construct (sub-construct and domain) using an excel sheet where each column represented each of the 14 papers, while each row gave an overview of all texts coded within one construct (sub-construct and domain). The coding spreadsheet is accompanied by a separate word document of compiled ‘notes’ which give more insight into the coding process (from the initial draft of framework analysis, to the final version), reasons for particular classifications and initial reflections on the analysis as recommended when conducting framework analysis [2].

References

1. Malterud K. Qualitative metasynthesis: A research method for medicine and health sciences. Abingdon, Oxon, New York, NY: Routledge; 2019.

2. Gale NK, Heath G, Cameron E, Rashid S, Redwood S. Using the framework method for the analysis of qualitative data in multi-disciplinary health research. BMC Med Res Methodol. 2013;13:117. doi:10.1186/1471-2288-13-117.
