# Supplementary material for: Barriers and facilitators to implementation of direct fruit and vegetables provision interventions in kindergartens and schools: a qualitative systematic review applying the consolidated framework for implementation research (CFIR)
Source: Int J Behav Nutr Phys Act. 2022 Jan 31;19:11. doi: 10.1186/s12966-022-01246-8 (PMC8805256; doi:10.1186/s12966-022-01246-8)
Supplement: Supplementary file 2 — Additional file 2. Definitions and Coding Clarifications [file 12966_2022_1246_MOESM2_ESM.docx]

**Additional file 2: Definitions and Coding Clarifications**

**Definitions (as directly applicable in the context of this paper):**

| **Direct provision of fruit and vegetable intervention (as stated in Protocol: registration number** CRD42020167697) | An intervention which would promote the intake of fresh and unprocessed fruit and vegetables (fruit and vegetable beverages are not included) by children, free of charge or subsidized, in kindergartens, primary and secondary school environments. Interventions which provide fresh fruit and vegetables on school property, at any time during the school day are included: 1) the provision (free or subsidised) of fruit and vegetables in classrooms, 2) provision (free or subsidised) of fruit and vegetables in cafeteria, school yard and elsewhere within school property (ex. vending machines), 3) the provision (free or subsidised) of fruit and vegetables –outside of usual school meals and/or during usual school meals. Intervention should ideally not be shorter than four weeks. |
| --- | --- |
| **Program/intervention** | ‘Program’ may be used in the place of ‘intervention’, when the intervention is referred to as such in the original source document. For definition of ‘intervention’ refer to Policy Evaluation Network (PEN) Glossary. |
| **(Intervention) component** | Core elements of intervention design. |
| **(Intervention) actions** | The actions undertaken when putting intervention components into practice. |
| **Children/student** | Up to age of 18. As some papers use the term ‘student’, this may also be used. |
| **Target group** | The group toward whom the intervention impact is aimed- in the context of this study, the primary target group are children, secondary target group are parents. |
| **Implementation actors/implementers** | Persons that have any role at all in the implementation of the intervention. In the context of the current papers, most often refers to teachers, although it can also be school administration, school staff, farmers/producers/suppliers/distributors. |
| **Study/paper/article** | Used interchangeably, all referring to a singular peer reviewed journal based publication. Multiple publications may be based on the same intervention. |
| **Influencing factors/determinant** | Used interchangeably. For definition of ‘determinant’ look at PEN Glossary. |

**PEN Glossary of Definitions 2020 (applicable to this paper, and generally applied by PEN, full Glossary to be made public on PEN website: https://www.jpi-pen.eu/)**

| **Intervention** | An umbrella term which includes any policy, programme or environmental change (physical and/or social) used to promote specific health behaviours or goals. [1] |
| --- | --- |
| **Intervention Implementation** | Act of converting program objectives into actions through deployment of resources, policy changes, regulations including the coordination or supervision of activities in support of the planned interventions. [2] |
| **Determinant** | Factors believed or empirically shown to have a causal effect on **implementation processes** and/or **implementation outcomes**, which may be targeted in order to achieve desired changes in individual, setting, or system. This may include barriers (or hinders, impediments), facilitators (enablers) or other contextual determinants. [3] |
| **Implementation Outcomes** | Under implementation evaluation, implementation outcomes are the effects of deliberate and purposive actions to implement new treatments, practices, and/or services. [4] |
| **Framework** | Frameworks are defined as a graphical or narrative representation of the key factors, concepts, or variables to explain the phenomenon under study, and as a minimum need to include the steps, strategies or factors relevant for the various stages of the development.  Frameworks may include evaluation implementation frameworks, determinant frameworks or conceptual frameworks. [5–7] |
| **Setting** | Refers to the specific environmental characteristics in which the actions are put into practice, including physical location or other policies implemented in the same time frame. [8–10] |
| **Context** | Set of circumstances or unique factors that surround a particular implementation effort.[9]  To better address implementation challenges in different settings, it is important to understand what happens when evidence-based practice (e.g. intervention) is 'woven together' with a team, department, or organisation. In literature, 'context', 'setting' and 'environment' are often used interchangeably. [3] |
| **Implementation Science** | Scientific study of methods to promote the systematic uptake of research findings and/or other evidence-based practices into routine practice, and hence, to improve the quality and effectiveness of health services. It includes the study of influences on healthcare professional and/or organizational behaviour. [11] |

**Coding Clarifications**

The definitions provided on the CFIR Website [12] for each construct, sub-construct were applied. Where additional clarification was needed, the CFIR Guide (available on the same website) was consulted.

Double coding was avoided. Where the same texts may be found under different constructs, it is only to provide the wider context so as to be able to fully understand the meaning. In these cases, the text coded uniquely under the particular construct is in bold.

| **Domain** | **Construct** | **Comment** |
| --- | --- | --- |
| **Intervention characteristics** |  | When looking at constructs under ‘intervention characteristics’, ‘outer setting’, characteristics of individuals’ and ‘process’, the content may reflect the views of the target group of the study, thus, can be teachers, but also farmers/producers/suppliers/distributors. |
|  | Intervention source | The single text under this construct could be double coded under ‘compatibility’ (implementation climate – inner setting domain). However, since the text implied a sense of ‘community ownership’ of farm to school, due to being compatible with community values – it was coded under this construct. |
|  | Evidence Strength and Quality |  |
|  | Relative Advantage |  |
|  | Adaptability |  |
|  | Trialability |  |
|  | Complexity |  |
|  | Design Quality and Packaging | Some of the text coded under ‘design quality and packaging’ could be double coded under ‘patient needs and resources’ as well as under ‘complexity’. |
|  | Cost | Text coded under ‘cost’ is distinguished from text coded under ‘external policy and incentives’ relating to financial need. When the text refers to allocation of internal funds, it is coded under the current construct. |
| **Outer setting** |  | When looking at constructs under ‘intervention characteristics’, ‘outer setting’, characteristics of individuals’ and ‘process’, the content may reflect the views of the target group of the study, thus, can be teachers, but also farmers/producers/suppliers/distributors. |
|  | Patient needs and resources | There were difficulties with some of the texts under this construct. They aim to show, that there is some inherent knowledge of the needs of children by those designing or implementing the intervention. And it usually pertains to the needs or resources independently of the content of the intervention. However, it is often difficult to make a determination, and some of the texts could possibly be double coded under ‘design quality and packaging’, ‘knowledge and beliefs about the intervention’. |
|  | Cosmopolitanism | Based on the definition of cosmopolitanism as provided in Damschroder et al., 2009 [9], the authors made the decision to include all text related to interactions between kindergarten/schools and farmers/producers/suppliers/delivery service persons – or any other actors external to the inner setting (kindergarten/school) under this construct .’ |
|  | Peer Pressure |  |
|  | External Policy and Incentives | Text coded under ‘cost’ is distinguished from text coded under ‘external policy and incentives’ relating to financial need. When the text refers to applying for, and allocation of external funds, it is coded under the current construct. |
| **Inner setting** |  | Contains information only pertaining to the ‘school’ setting. Thus, even if the paper reflected the views from the perspective of producers/suppliers/distributors, and their perspective may be incorporated in the other domains – even in the context of those papers, the primary setting has been the school. |
|  | Structural characteristics |  |
|  | Networks and communications |  |
|  | Culture |  |
|  | Implementation Climate (tension for change; compatibility; relative priority; organizational incentives and rewards; goals and feedback; learning climate) |  |
|  | Readiness for implementation (leadership engagement; available resources; access to knowledge and information) |  |
| **Characteristics of individuals** |  | When looking at constructs under ‘intervention characteristics’, ‘outer setting’, characteristics of individuals’ and ‘process’, the content may reflect the views of the target group of the study (different from target group of intervention), thus, can be teachers, but also farmers/producers/suppliers/distributors |
|  | Knowledge, beliefs about the intervention | There were difficulties in coding between ‘patient needs and resources’ and ‘knowledge and beliefs about the intervention’. Text under ‘patient needs and resources’ implies knowledge about needs of children, independently of the intervention. While text under ‘knowledge, beliefs about the intervention’ reflects perceived benefits to the target group of the intervention (children), by the implementers- most often teachers, due to the intervention.  For this reason, ‘perceptions of behavior change’ in the target group (children), expressed by the implementers (most often teachers) were coded here. However, ‘perceptions of behavior change’ expressed by the target group themselves (children) or their parents (secondary target group, external change agents) were not coded, as ‘characteristics of individuals’ pertains mainly to implementing actors. |
|  | Self-efficacy |  |
|  | Individual stage of change | Although none of the papers collected thorough information on Rodgers (2003) stages of change, some text was nonetheless coded here. The decision was made, as the text reflected some aspects of the different stages as they are described in the theory. However, it should be noted that this is superficial, and more thorough research is necessary on this construct. |
|  | Individual identification with organization |  |
|  | Other personal attributes |  |
| **Process** |  | When looking at constructs under ‘intervention characteristics’, ‘outer setting’, characteristics of individuals’ and ‘process’, the content may reflect the views of the target group of the study, thus, can be teachers, but also farmers/producers/suppliers/distributors |
|  | Planning |  |
|  | Engaging (opinion leaders; formally appointed internal implementation leaders; champions; external change agents) |  |
|  | Executing | Beliefs about the intervention are distinguished from perceptions about the execution of the intervention. Text coded under ‘execution’ refers to the consequence for implementation of executing a particular component of the intervention. |
|  | Reflecting and evaluating |  |

References

1. Woods CB, Volf K, Kelly L, Casey B, Gelius P, Messing S, et al. The evidence for the impact of policy on physical activity outcomes within the school setting: A systematic review. J Sport Health Sci 2021. doi:10.1016/j.jshs.2021.01.006.

2. Green LW, Kreuter MW. Health program planning: An educational and ecological approach. 4th ed. Boston: McGraw-Hill; 2005.

3. Nilsen P, Bernhardsson S. Context matters in implementation science: a scoping review of determinant frameworks that describe contextual determinants for implementation outcomes. BMC Health Serv Res. 2019;19:189. doi:10.1186/s12913-019-4015-3.

4. Proctor E, Silmere H, Raghavan R, Hovmand P, Aarons G, Bunger A, et al. Outcomes for implementation research: conceptual distinctions, measurement challenges, and research agenda. Adm Policy Ment Health. 2011;38:65–76. doi:10.1007/s10488-010-0319-7.

5. Moullin JC, Sabater-Hernández D, Fernandez-Llimos F, Benrimoj SI. A systematic review of implementation frameworks of innovations in healthcare and resulting generic implementation framework. Health Res Policy Syst. 2015;13:16. doi:10.1186/s12961-015-0005-z.

6. Nilsen P. Making sense of implementation theories, models and frameworks. Implement Sci. 2015;10:53. doi:10.1186/s13012-015-0242-0.

7. Rycroft-Malone J, Bucknall T. Models and Frameworks for Implementing Evidence-Based Practice: Linking Evidence to Action. 1st ed. Hoboken: Wiley; 2013.

8. Bronfenbrenner U. The ecology of human development: Experiments by nature and design. Cambridge, MA: Harvard University Press; 1979.

9. Damschroder LJ, Aron DC, Keith RE, Kirsh SR, Alexander JA, Lowery JC. Fostering implementation of health services research findings into practice: a consolidated framework for advancing implementation science. Implement Sci. 2009;4:50. doi:10.1186/1748-5908-4-50.

10. Rutter H, Savona N, Glonti K, Bibby J, Cummins S, Finegood DT, et al. The need for a complex systems model of evidence for public health. The Lancet. 2017;390:2602–4. doi:10.1016/S0140-6736(17)31267-9.

11. Eccles MP, Mittman BS. Welcome to Implementation Science. Implementation Sci 2006. doi:10.1186/1748-5908-1-1.

12. Consolidated Framework for Implementation Research (CFIR) guide. https://cfirguide.org/.
