# Supplementary material for: Barriers and facilitators to implementation of direct fruit and vegetables provision interventions in kindergartens and schools: a qualitative systematic review applying the consolidated framework for implementation research (CFIR)
Source: Int J Behav Nutr Phys Act. 2022 Jan 31;19:11. doi: 10.1186/s12966-022-01246-8 (PMC8805256; doi:10.1186/s12966-022-01246-8)
Supplement: Supplementary file 4 — Additional file 4. Quality assessment process notes [file 12966_2022_1246_MOESM4_ESM.docx]

**Notes on process of quality assessment**

- From the very outset, there has been a complete agreement on number of voted ‘yes’ and ‘no’ on 9 out of the 14 papers – papers 2, 4, 5, 7, 8, 10, 12, 13, 14
- The remaining there was usually a 1 vote difference (in all cases BM had a ‘no’ vote where MS had voted ‘yes’)

**Overview of disagreements:**

Paper 1 disagreements:

-research design (BM NO, MS YES)

-references (BM NO, MS YES)

Paper 3 disagreements:

-data analysis (BM NO, MS YES)

-references (BM NO, MS YES)

Paper 6 disagreements:

-reflexivity (BM NO, MS YES)

Paper 9 disagreements:

-value of the research (BM NO, MS YES)

Paper 11 disagreements:

-references (BM NO, MS YES)

**Additional:**

Paper 8, overall number of YES and NO same by both reviewers, but disagreements within:

-theoretical framework (BM NO, MS YES)

-analysis (MS NO, BM YES)

**Overall common (MS, BM) assessment:**

- Overall, we found papers to be especially weak when it came to **reflexivity** (assessment question 6) and **theoretical framework** (assessment question 8) across the board.
- In terms of reflexivity, 9 out of the 14 studies did not discuss it sufficiently or at all according to BM assessment, and 8 out of the 14 studies did not discuss it sufficiently or at all according to MS assessment
- In terms of theoretical framework, it was found lacking in 12 out of the 14 studies according to BM assessment, and 11 out of the 14 studies according to MS assessment
- In addition, both BM and MS had concerns in terms of the ethical considerations of two papers (12, 13). In paper 12, the authors explore the relationship between school culture and farm to school programs, but are employed by a farm (and this is not explored or reflected upon as a potential conflict of interest). In paper 13, study approval was granted by the institutional board of the agency funded to complete the implementation evaluation.

**Conclusion:**

As most papers were evaluated positively (receiving a ‘YES’ in at least 9 out of the 12 assessment questions), we do not recommend exclusion of any of the papers up to this point, based on the quality assessment (one paper only had 7 ‘YES’ assessments, 5 ‘NO’ by BM and 8 ‘YES’, 4 ‘NO’ by MS).
